# Public Health und Pflegewissenschaft: Zwei Disziplinen mit gemeinsamen Zielen

**DOI:** 10.1007/s00103-023-03692-6

**Published:** 2023-05-05

**Authors:** Florian Fischer, Joseph Kuhn, Adelheid Kuhlmey

**Affiliations:** 1 Bayerisches Zentrum Pflege Digital, Hochschule für angewandte Wissenschaften Kempten, Albert-Einstein-Straße 6, 87437 Kempten, Deutschland; 2 Institut für Public Health, Charité – Universitätsmedizin Berlin, Charitéplatz 1, 10117 Berlin, Deutschland; 3 GP1 Gesundheitsberichterstattung, Epidemiologie, Sozialmedizin, Bayerisches Landesamt für Gesundheit und Lebensmittelsicherheit, Veterinärstr. 2, 85764 Oberschleißheim, Deutschland; 4 Institut für Medizinische Soziologie und Rehabilitationswissenschaft, Charité – Universitätsmedizin Berlin, Charitéplatz 1, 10117 Berlin, Deutschland

Etwa 30 Jahre sind seit der Gründungsphase sowohl von Public Health als auch der Pflegewissenschaft in Deutschland vergangen. Die Konstituierung bzw. Etablierung dieser Disziplinen ging mit einem im internationalen Vergleich großen Nachholbedarf einher und stellte in gewisser Hinsicht ein Projekt nachholender Modernisierung nach angloamerikanischen Vorbildern dar [1, 2]. Die eingeschlagenen Entwicklungswege dieser beiden Disziplinen in Bezug auf Forschung, Lehre und Praxis waren gleichwohl unterschiedlich und wurden auch kritisch reflektiert [2]. Im Kern ging es jedoch zunächst um eine jeweils fachspezifische Profilierung und damit einhergehende Abgrenzung voneinander.

Zwischen Public Health und Pflegewissenschaft bestehen große Schnittmengen – insbesondere in den Bereichen der Versorgungsforschung und -gestaltung sowie Prävention und Gesundheitsförderung [3]. Ebenso zeigt sich mit Blick auf die übergreifenden Zielsetzungen, dass sowohl Public Health als auch Pflege sämtliche unterstützenden Maßnahmen und Handlungen umfassen, die der Erhaltung, Wiederherstellung oder Anpassung von physischen, psychischen und sozialen Funktionen und Aktivitäten des alltäglichen Lebens dienen – und somit letztlich Lebensqualität, Wohlbefinden und Gesundheit fördern. Daher sind Ansätze von Public Health auch der Pflege immanent und die Pflege ist zugleich ein Handlungsbereich für Public-Health-Interventionen.

Die Bedeutung des Zusammenwirkens der Perspektiven und Expertisen aus beiden Disziplinen – und damit verbunden auch der zugehörigen Professionen – ist eigentlich schon seit langer Zeit bekannt, wird aber besonders deutlich, wenn langfristige Transformationsprozesse (z. B. demografischer Wandel, Digitalisierung) und auch aktuell einwirkende Krisen (z. B. COVID-19-Pandemie, Klimawandel, kriegerische Auseinandersetzungen in der Ukraine) betrachtet werden, welche sich wechselseitig beeinflussen.

In der Pflege spiegeln sich viele gesellschaftliche Megatrends wider: der Gewinn an Lebenserwartung, der trotz aller Konjunktur von Prävention und Gesundheitsförderung nicht nur ein Gewinn an gesunden Lebensjahren ist, die Veränderungen der Sozialstrukturen mit einer Zunahme von Alleinlebenden, die Migrationsbewegungen der letzten Jahrzehnte, die zunehmend auch interkulturelle Anforderungen an die Pflege stellen, oder die Ökonomisierung des Gesundheitswesens, die den „Pflegemarkt“ prägt und ihre eigenen Folgeprobleme mit sich bringt.

Deutschland gehört wie viele Industrieländer zu den Gesellschaften des langen Lebens. Im Jahr 2021 waren 22 % der Bevölkerung 65 Jahre oder älter, 7,3 % sogar über 80 Jahre [4]. Die meisten der Älteren sind zwar bei angemessen guter Gesundheit und können selbstbestimmt und eigenständig leben, aber im Altersgang nehmen Einbußen der körperlichen und geistigen Gesundheit zu und es erhöht sich das Risiko einer Pflegebedürftigkeit. Zusätzlich zu den offiziell vorliegenden Zahlen zur Pflegebedürftigkeit besteht eine hohe Dunkelziffer, da nicht alle Menschen, die auf Unterstützung im Alltag angewiesen sind, Leistungen der Pflegeversicherung in Anspruch nehmen. Dieser Zusammenhang zwischen der Alterung der Bevölkerung und den gesundheitlichen Versorgungsnotwendigkeiten wird in den kommenden Jahrzehnten in Deutschland durch die Vertreter:innen der Babyboomer-Generation eine besondere Dynamik erreichen, wenn diese aus dem Erwerbsleben ausscheiden und potenziell auch Unterstützungsbedarfe entwickeln. Dies fordert alle Akteure im Gesundheitssystem heraus, so insbesondere auch die Pflege.

In den letzten Jahren hat sich das pflegerische Angebot zwar erkennbar ausdifferenziert, auch die Unterstützung für pflegende Angehörige ist besser geworden, für Berufstätige gibt es mehr Möglichkeiten, Beruf und Pflege zu vereinbaren. Aber gesellschaftlich nachhaltige Antworten darauf, wie ein würdiges Altern für alle, auch für sozial Schwache, aussehen soll, wenn die eigenen Kräfte nachlassen, stehen ebenso aus wie die Zukunftsgestaltung des Pflegeberufes und -systems. Selbst im Kernbereich der institutionalisierten Pflege, den Pflegeheimen, sind grundlegende Fragen ungeklärt – wie die Situation der Heimbewohner:innen in den Zeiten der COVID-19-Pandemie dramatisch aufzeigte.

Hoffnungen werden unter anderem in technische Lösungen gesetzt, die ebenfalls neue Märkte versprechen. Digitale Technologien finden vermehrt Anwendung, um z. B. die Pflegedokumentation zu vereinfachen oder auch einen Austausch zwischen verschiedenen Akteuren zu erleichtern. Zugleich wurde das Bedürfnis nach persönlicher, emotionaler und sozialer Zuwendung in der Altenpflege während der COVID-19-Pandemie überdeutlich. Auch die prekäre Situation der Pflegekräfte, wenn auch bereits bekannt und seit einigen Jahren auf der gesundheitspolitischen Agenda, wurde in der Öffentlichkeit noch einmal wesentlich deutlicher. Die Infektionsschutzmaßnahmen in den Pflegeheimen, etwa die zeitweise strikten Besuchsverbote, haben deutliche Spuren hinterlassen und Grundfragen der Pflegeethik und der Pflegequalität aufgeworfen. Trotz ehrlich gemeinter öffentlicher Anerkennung, auch in Bezug auf das Versprechen von Verbesserungen der Arbeitsbedingungen, scheint es trotz der jüngsten Diskussionen um eine längst überfällige Pflegereform (in Bezug auf Fragen der Finanzierung und Leistungsdynamisierung) insgesamt noch keinen Durchbruch zu einer „neuen Pflege“ zu geben.

Somit wird es wohl zunächst bei den kleinen Schritten für ein großes Thema, für ein Thema mit Relevanz für Public Health, bleiben. Das vorliegende Heft versammelt Beiträge, die den Public-Health-Charakter vieler Bereiche der Pflege deutlich machen sollen. Es nimmt eine bevölkerungsbezogene und interdisziplinäre Perspektive auf Pflege und pflegerische Versorgungsforschung ein. Dabei geht es zentral um die Altenpflege. Die thematischen Schwerpunkte der Beiträge enthalten sowohl Übersichts- und Diskussionsbeiträge als auch Originalarbeiten, um einen Einblick in das Themenfeld aus verschiedenen Blickwinkeln zu geben. Im Vordergrund stehen dabei (soziale) Aspekte für verschiedene Zielgruppen (Pflegebedürftige, informell sowie professionell Pflegende) in diversen Settings. Auch Möglichkeiten der Prävention, Reformbedarfe und insbesondere aktuelle Entwicklungen (z. B. in Bezug auf Digitalisierung) sollen aufgezeigt und diskutiert werden. Insgesamt soll dieses Themenheft somit als Impuls dienen, um zukünftig ein Bündnis beider Disziplinen stärker voranzubringen, welches zwar die jeweiligen Autonomieansprüche wahrt, aber eben auch zu einer Verbesserung der Bevölkerungsgesundheit beiträgt.
